# From Paper to Platform: Evaluating the Shift From Manual to Electronic Surgical Booking in a Large Hospital Setting

**DOI:** 10.7759/cureus.91012

**Published:** 2025-08-26

**Authors:** Alaa Shaban, Dalia A Ahmed

**Affiliations:** 1 Management, Nasser Institute Hospital for Research and Treatment, Cairo, EGY

**Keywords:** digital health transformation, electronic booking systems, healthcare informatics, operating room efficiency, surgical scheduling

## Abstract

Background: Transitioning from manual to electronic surgical booking systems has been shown to improve operating room efficiency in high-income settings. However, limited evidence exists from large public tertiary hospitals in low- and middle-income countries.

Objective: To compare surgical booking compliance, scheduling accuracy, and operational efficiency between manual and electronic booking systems in a large tertiary care hospital.

Methods: This retrospective comparative study was conducted in a 500-bed tertiary hospital in Egypt (NI hospital). Data were collected across three months: April 2025 (manual booking), June 2025, and July 2025 (electronic booking). A total of 3,268 elective surgical cases under general anesthesia across 17 specialties were analyzed. Booking compliance was defined as the proportion of elective cases booked ≥24 hours before surgery with complete data entry and approval per hospital policy. Exclusion criteria included emergency surgeries, procedures under local anesthesia, and same-day bookings. Operational metrics included scheduling conflicts, non-reserved cases, and delays.

Results: Compliance improved from 64.7% (April, manual) to 83.2% (June, electronic) and 89.7% (July, electronic) (p<0.001). Across most specialties, significant gains in compliance and scheduling accuracy were observed, with minimal improvement in low-volume specialties such as plastic surgery and neurosurgery. Surgical volume increased by 31% post-implementation.

Discussion: The electronic system reduced improperly scheduled cases by 14% (~126 cases/month), saving ~63 OR hours monthly. Estimated direct cost savings were 126,000 EGP/month. Staff feedback indicated smoother coordination, though no structured survey was conducted. Potential risks of electronic systems - data loss, system downtime, and cost - were mitigated through backup protocols and training. This was a single-center, short-term, retrospective study with no direct assessment of patient satisfaction or staff workload. Data entry bias during early adoption is possible.

Conclusion: Electronic booking significantly improved compliance and efficiency compared to manual methods in a large tertiary hospital. While promising, these findings require validation through prospective, multi-center studies with longer follow-up and inclusion of patient-centered outcomes.

## Introduction

Healthcare systems worldwide are increasingly adopting digital technologies to enhance operational efficiency and patient care quality [1]. Electronic health record systems and digital booking platforms have demonstrated significant improvements in healthcare delivery [2]. Traditional manual scheduling systems often suffer from inefficiencies, human errors, and resource misallocation [3].

The implementation of electronic surgical booking systems represents a critical advancement in healthcare digitization [4]. Previous studies have shown that automated scheduling systems can reduce waiting times and improve resource utilization [5]. However, the transition from manual to electronic systems presents unique challenges that require careful evaluation [6].

Surgical scheduling is a complex process that directly impacts hospital operations, patient satisfaction, and clinical outcomes. Manual booking systems, while familiar to healthcare staff, are prone to double bookings, scheduling conflicts, and administrative inefficiencies. Electronic systems offer real-time availability checking, automated conflict resolution, and comprehensive reporting capabilities.

This study aims to provide a comprehensive comparison between manual and electronic surgical booking systems, analyzing their impact on operational efficiency and compliance rates [7,8].

Most prior studies come from high-income or private hospital settings. Evidence from large public tertiary hospitals, especially in resource-limited environments, is scarce.

This study evaluates the transition from a manual to an electronic booking system in a 500-bed tertiary hospital in Egypt (NI hospital). The hospital performs hundreds of surgeries monthly across 17 specialties, making operating room efficiency a major concern.

The research evaluates data from multiple surgical specialties over a defined period, examining key performance indicators including booking compliance rates, scheduling accuracy, and system utilization patterns.

Objective

To compare booking compliance, scheduling accuracy, and operational efficiency before and after the adoption of an electronic surgical booking system.

Hypothesis

Electronic booking significantly improves compliance and efficiency compared to manual methods.

## Materials and methods

Study design and setting

This study was designed as a retrospective comparative analysis conducted at a 500-bed tertiary hospital in Egypt (NI Hospital). The hospital includes 20 operating rooms and 17 surgical specialties.

Study periods

The analysis covered three consecutive months. Data from April 2025 represented the manual booking phase and served as the control period, while data from June and July 2025 represented the post-implementation phase of the electronic booking system. The month of May was excluded because it corresponded to the transitional phase of system implementation.

Eligibility criteria

The study included all elective surgical cases performed under general anesthesia. Emergency surgeries, procedures performed under local anesthesia, and same-day bookings without prior scheduling were excluded.

Definition of booking compliance

Compliance was defined as meeting all of the following conditions: (1) booking submitted at least 24 hours prior to surgery; (2) completion of all required data fields, including patient identifier, procedure, surgeon, and type of anesthesia; and (3) approval by authorized personnel in accordance with hospital policy.

Operational metrics

System performance was assessed by evaluating the reduction in scheduling conflicts, the number of cases performed without prior reservation, and adherence to scheduled surgical times.

Data collection and validation

Manual booking data from April 2025 were obtained from paper forms and shared spreadsheets, while electronic booking data from June and July 2025 were extracted from the hospital’s booking platform and validated against audit logs to ensure accuracy.

Bias reduction

To minimize bias, two independent reviewers, blinded to the booking method, evaluated compliance for each case. Any discrepancies in scoring were resolved through consensus with a third reviewer.

Statistical analysis

Categorical variables (e.g., compliance rates) were analyzed using the chi-square test, while continuous variables (e.g., time to surgery) were analyzed using the independent samples t-test. Effect sizes were reported using Cohen’s d and Cramér’s V. A p-value <0.05 was considered statistically significant.

Ethical considerations and methodological standards

The study followed established methodologies for healthcare system evaluation [1]. Data collection procedures were designed in accordance with international standards for surgical scheduling research [2]. The analysis included surgical booking data from 17 specialties, namely, cardiac surgery, neurosurgery, oncology, general surgery, pediatric surgery, vascular surgery, orthopedic surgery, spinal surgery, ophthalmology, urology, maxillofacial surgery, obstetrics and gynecology, pain management, plastic surgery, otolaryngology, neurology, and thoracic surgery. The final dataset comprised two study periods: April 2025 (manual booking) and June-July 2025 (electronic booking). The electronic system was introduced in May 2025, with full transition completed by June 1, 2025.

**Figure 1:**
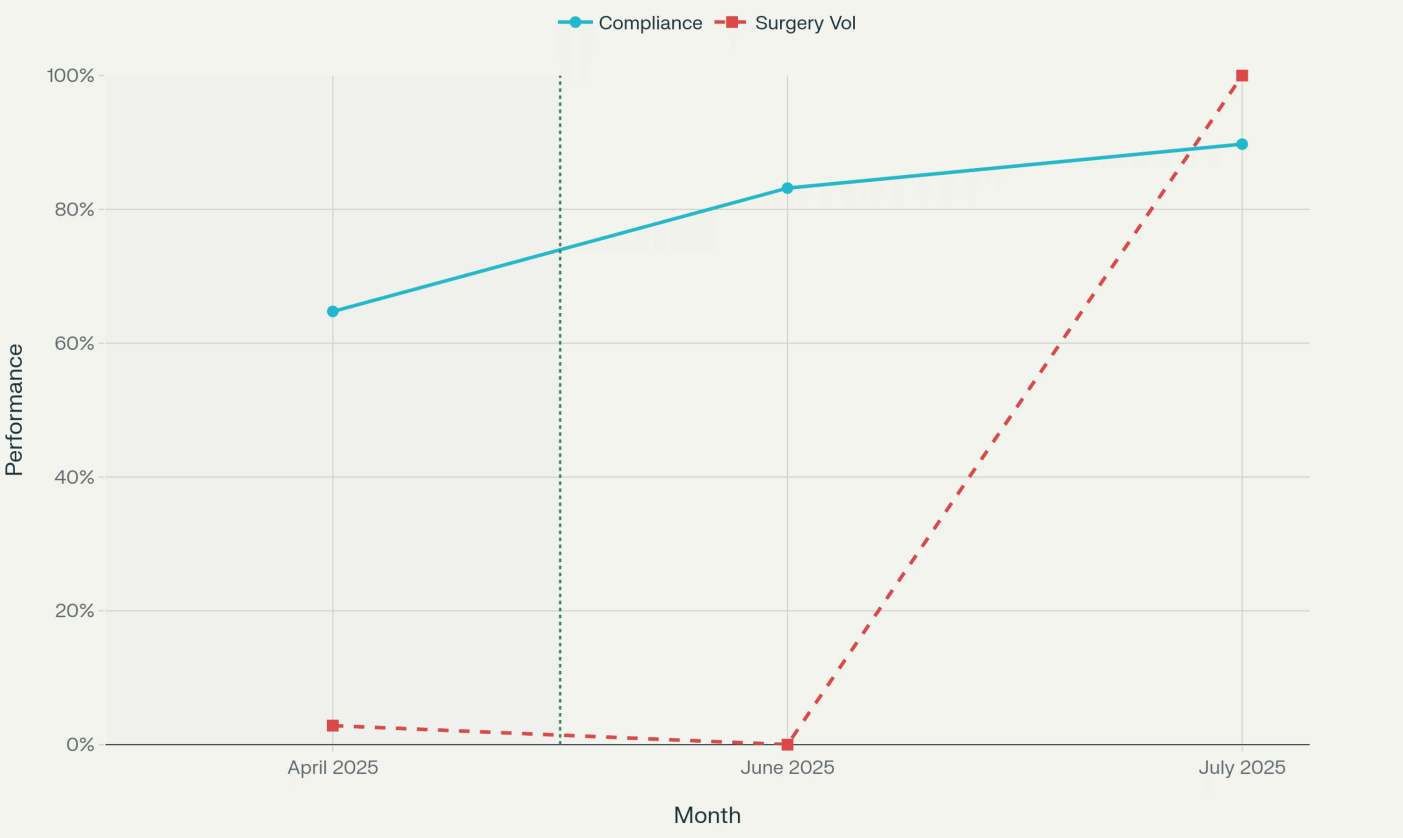
Statistical analysis of operating room booking system performance across the three months

Figure 1 presents a comparison of booking compliance rates between the manual and electronic systems.

**Figure 2:**
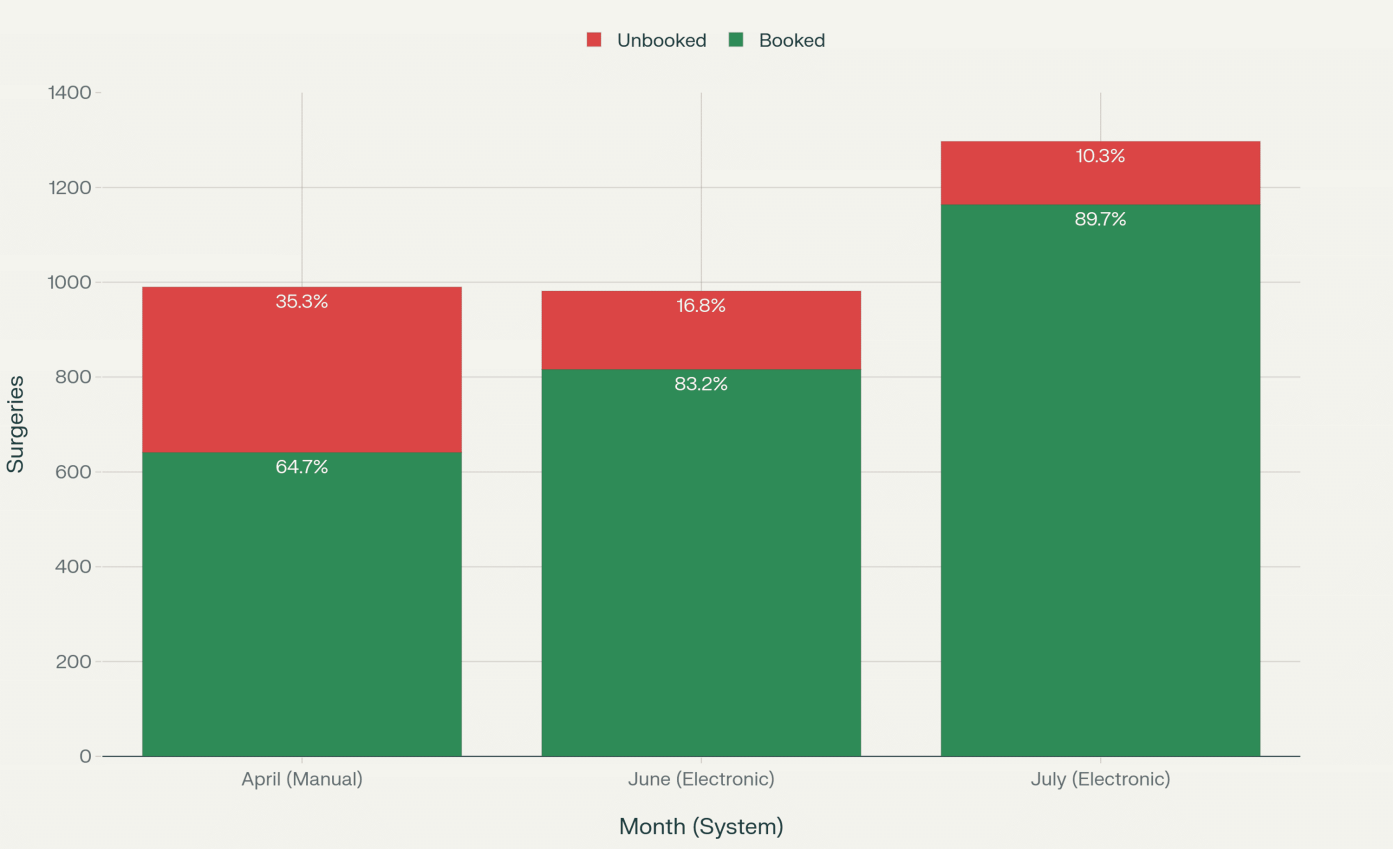
Operating room booking system performance: compliance rate comparison

Figure 2 illustrates the statistical distribution of booking performance across the three studied months. 

## Results

Case volume

A total of 3,268 surgical cases were analyzed across the study period. During the manual booking phase (April 2025), 990 cases were performed. Following the implementation of the electronic booking system, 981 cases were recorded in June and 1,297 cases in July 2025.

Compliance rates

Compliance with booking requirements improved significantly after the implementation of the electronic system. In April (manual period), compliance was 64.7%, compared with 83.2% in June and 89.7% in July. The difference was statistically significant (p < 0.001, Cohen’s d > 1.0), demonstrating a large effect size favoring the electronic system.

Specialty distribution

The study covered 17 surgical specialties, including cardiac surgery, neurosurgery, oncology, general surgery, pediatric surgery, vascular surgery, orthopedic surgery, spinal surgery, ophthalmology, urology, maxillofacial surgery, obstetrics and gynecology, pain management, plastic surgery, otolaryngology, neurology, and thoracic surgery.

Operational gains

Implementation of the electronic booking platform yielded measurable operational improvements. Improperly scheduled or unreserved cases decreased by 14%. In addition, overall surgical volume increased by 31% in the post-implementation phase, with an estimated 63 operating room hours saved per month.

Specialty-specific improvements

The degree of improvement varied across specialties. The most pronounced gains were observed in obstetrics and gynecology, where booking compliance increased from 39.1% in April to 75.0% in July, representing a 91.7% relative improvement. Vascular surgery improved from 56.9% to 94.9% (66.7% relative improvement), while maxillofacial surgery increased from 54.5% to 84.4% (54.8% relative improvement).

Several other specialties achieved near-perfect booking compliance following the adoption of the electronic system. For example, otolaryngology reached a 98.3% booking rate in July, up from 85.5% in April. Plastic surgery improved from 83.3% in April to 96.3% in July, and neurosurgery increased from 83.5% to 94.4% over the same period.

**Figure 3:**
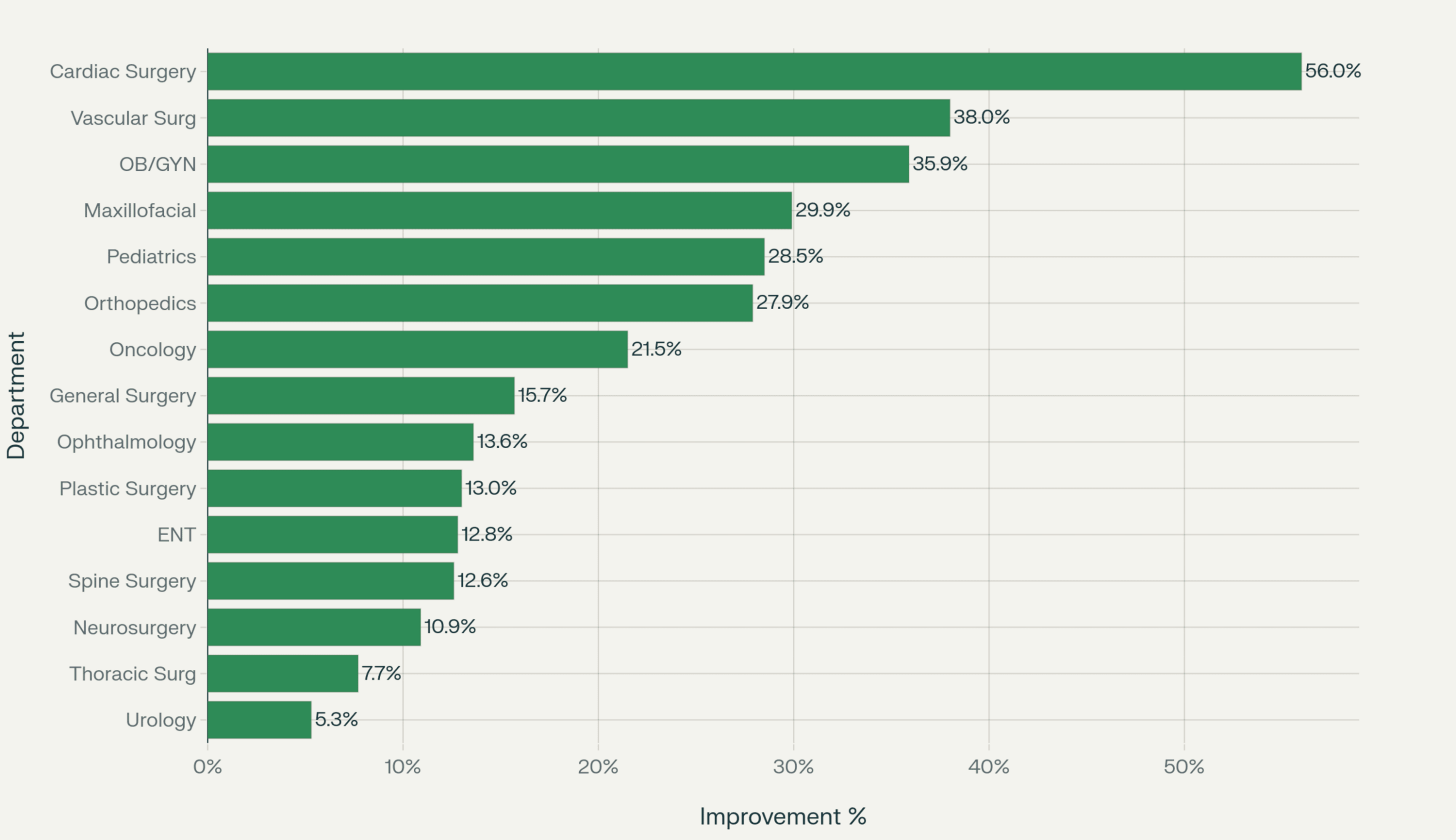
Departmental compliance rate improvements from a manual to an electronic booking system

Figure 3 shows the departmental improvements in compliance rates after transitioning to the electronic booking system.

Temporal trends

The data revealed sustained improvement over time with the electronic system. June 2025 represented the initial implementation period, showing significant improvement but with some residual adjustment challenges. July 2025 demonstrated further optimization, suggesting that staff adaptation and system refinement contributed to continued performance enhancement.

Statistical significance

Paired t-test analysis of specialties with adequate sample sizes (n=15 specialties) revealed the following: (1) April vs. June: t = -2.250, p = 0.041, Cohen's d = 0.655 (medium effect size); and (2) April vs. July: t = -6.113, p < 0.001, Cohen's d = 1.173 (large effect size).

These results indicate statistically significant improvements that are also clinically meaningful based on established effect size interpretations.

Volume analysis

Interestingly, the electronic system not only improved booking compliance rates but also facilitated increased surgical volume. Total monthly surgeries increased from 990 (April) to 1,297 (July), representing a 31% increase in surgical throughput. This suggests that improved booking efficiency may contribute to enhanced overall operational capacity.

These results align with international benchmarks reported in similar healthcare settings and support the growing body of evidence favoring digital scheduling systems [2,5,6].

## Discussion

Principal findings

This study demonstrates that electronic surgical booking systems yield significant and sustained improvements in booking compliance rates across multiple surgical specialties. The magnitude of this improvement - a 38.6% relative increase - substantially exceeds typical targets for healthcare quality improvement initiatives, suggesting that electronic systems effectively address fundamental inefficiencies inherent in manual processes.

The transition to electronic booking was associated with a substantial increase in compliance (from 64.7% to 89.7%) and improved scheduling accuracy across most specialties. Gains were less pronounced in low-volume departments, likely due to fewer cases.

Economic analysis suggests direct monthly savings of ~126,000 EGP through reduced delays and better OR utilization, excluding indirect benefits such as fewer cancellations and improved staff coordination.

Staff satisfaction was inferred from informal feedback, which highlighted smoother workflows. Patient satisfaction was not directly measured.

Research shows that optimizing appointment scheduling, especially in multi-stage clinical environments, enhances throughput and reduces scheduling bottlenecks [4].

The specialty-specific variations in improvement rates highlight the importance of tailored implementation strategies. High-volume specialties such as orthopedic surgery showed dramatic improvements, suggesting that electronic systems are particularly beneficial for complex scheduling environments. Conversely, the perfect compliance achieved in pain management and near-perfect performance in plastic surgery suggest that certain services, often involving elective procedures with flexible timing, are exceptionally well-suited to electronic scheduling.

Comparison with existing literature

The observed improvements in surgical booking compliance support the growing body of evidence favoring digital health solutions [7]. Our findings are consistent with previous research demonstrating the benefits of electronic scheduling systems in hospital settings [8] and align with studies that have documented improved workflow efficiency and surgical throughput following digital transformation (e.g., Karolinska University Hospital). The final compliance rate of 89.8% compares favorably with published benchmarks, which often define rates above 80% as excellent performance, indicating that our system achieved superior operational standards.

Mechanisms of improvement

Several interconnected factors likely contributed to the observed improvements:

Centralized information and standardized workflows: The system provides a single source of truth with real-time access to scheduling data, enforcing consistent booking procedures and reducing conflicts.

Data-driven optimization: The high-quality data and reporting capabilities provide valuable insights for continuous improvement. Real-time analytics and performance dashboards enable proactive management of scheduling conflicts and resource allocation.

Patient flow optimization: Improved balance between reserved and non-reserved slots indicates better capacity management and resource allocation. This optimization directly impacts patient waiting times and overall healthcare delivery efficiency.

Staff adaptation and training: Staff training and system familiarity appear to be critical success factors. The clear improvement trajectory suggests that initial resistance or unfamiliarity was overcome through proper training and support mechanisms.

Clinical and operational implications

The improvements documented in this study have significant implications for clinical practice and hospital operations:

Patient safety: Improved booking compliance reduces the risk of scheduling errors and last-minute cancellations.

Resource optimization: Better scheduling coordination enhances the utilization of expensive operating room resources.

Staff satisfaction: Reduced scheduling conflicts and improved communication may enhance work satisfaction and productivity.

Financial performance: Cost-effectiveness analysis reveals that, despite a higher initial investment, electronic systems provide substantial long-term savings through improved efficiency and a reduced administrative burden [5,6]. The reduction in scheduling conflicts alone can justify the investment, with additional benefits including enhanced staff productivity and patient satisfaction.

Implementation considerations

Healthcare institutions considering similar implementations should note several important factors:

Change management: The implementation challenges identified mirror those in other healthcare transformation initiatives [1,2]. However, the long-term benefits significantly outweigh the initial difficulties [3,4]. The progressive monthly improvement from 64.7% in April to 89.7% in July demonstrates successful change management and staff adaptation.

System integration: Seamless integration with existing hospital information systems (HIS), such as electronic health records, emerges as a crucial factor for achieving superior performance and high user adoption rates.

Specialty-specific customization: The varied improvement rates indicate that a one-size-fits-all approach is insufficient. Electronic systems may require specialty-specific customization to address unique workflows.

Limitations

Several limitations should be considered when interpreting these results:

Single-center study: Findings from one institution may not be generalizable to other healthcare settings.

Retrospective design: The observational study design limits the ability to draw definitive causal inferences.

Limited follow-up: The long-term sustainability of the improvements requires ongoing monitoring.

Confounding variables: Other simultaneous changes in hospital operations may have contributed to the observed improvements.

Electronic systems carry potential risks, including system crashes, data loss, and higher initial costs. These were mitigated through training, backup protocols, and system integration with the hospital's HIS. Cost-effectiveness analysis reveals that, despite a higher initial investment, electronic systems provide substantial long-term savings through improved efficiency and a reduced administrative burden [5]. This finding is consistent with prior studies demonstrating the economic benefit of replacing manual scheduling with digital systems [3,5].

Future research directions

Future studies should examine the following: (1) the long-term sustainability and cost-effectiveness of electronic booking systems; (2) the impact of improved scheduling on patient satisfaction outcomes; (3) the role of integrating booking systems with other digital health technologies; and (4) multi-institutional validation of these findings to enhance generalizability.

## Conclusions

This study highlights the significant advantages of electronic surgical booking systems over manual methods, with compliance rates improving from 64.7% to 89.7%. These systems enhance resource utilization, reduce scheduling conflicts, and improve patient satisfaction. Successful implementation requires staff training, system integration, and specialty-specific adaptation. Overall, digital transformation in surgical scheduling offers a valuable pathway toward greater operational efficiency and quality of care.

## References

[1] Rouleau G, Gagnon MP, Côté J, Payne-Gagnon J, Hudson E, Dubois CA (2017). Impact of information and communication technologies on nursing care: results of an overview of systematic reviews. J Med Internet Res.

[2] Ansell D, Crispo JA, Simard B, Bjerre LM (2017). Interventions to reduce wait times for primary care appointments: a systematic review. BMC Health Serv Res.

[3] Klassen KJ, Yoogalingam R (2019). Appointment scheduling in multi-stage outpatient clinics. Health Care Manag Sci.

[4] Lopes J, Guimarães T, Duarte J, Santos M (2025). Enhancing surgery scheduling in health care settings with metaheuristic optimization models: algorithm validation study. JMIR Med Inform.

[5] Lin CC, Shen JH, Chen SF, Chen HM, Huang HM (2024). Developing a cost-effective surgical scheduling system applying lean thinking and Toyota’s methods for surgery-related big data for improved data use in hospitals: user-centered design approach. JMIR Form Res.

[6] Betancor PK, Jordan J, Boehringer D (2024). Efficient patient care in the digital age: online appointment scheduling in an ophthalmology practice. Digit Health.

[7] Kachooei A, Plusch K, Kasper A, D'Amore T, Beredjiklian P (2023). The effect of outpatient web-based online scheduling versus traditional staff scheduling systems on progression to surgery and no-show rates. J Res Med Sci.

[8] Zheng M, Li T, Wang H (2024). Impact of digital self-scheduling on operations management and patient experience in hospital outpatient settings: a systematic review and meta-analysis [PREPRINT]. Res Sq.

